# Adaptations and Heterogeneity of Treatment Effects in Platform Trials—Protocol for Two Methodological Studies

**DOI:** 10.1111/aas.70044

**Published:** 2025-05-07

**Authors:** Tine Sylvest Meyhoff, Aksel Karl Georg Jensen, Anders Perner, Ewan C. Goligher, Marion K. Campbell, Morten Hylander Møller, Anders Granholm

**Affiliations:** ^1^ Department of Intensive Care Copenhagen University Hospital – Rigshospitalet Copenhagen Denmark; ^2^ Section of Biostatistics, Department of Public Health University of Copenhagen Copenhagen Denmark; ^3^ Department of Clinical Medicine University of Copenhagen Copenhagen Denmark; ^4^ Interdepartmental Division of Critical Care Medicine University of Toronto Toronto Canada; ^5^ Aberdeen Centre for Evaluation University of Aberdeen Aberdeen UK

**Keywords:** adaptive analyses, adaptive platform trial, heterogeneity of treatment effects

## Abstract

**Background:**

Adaptive platform trials bring opportunities for improved infrastructure and effective advancement in medical care but are methodologically complex. Assessment of heterogeneity of treatment effects (HTE) according to participant characteristics and adaptations, including adaptive stopping, are important methodological features in these trials, which may be approached in multiple ways. We aim to characterise the assessment of HTE and use of adaptations, including their key methodological features, in adaptive platform trials.

**Methods:**

This protocol outlines two methodological studies, which will be based on a common, systematic literature search and data extraction. We will include adaptive platform trials conducted from 2005 onwards. Screening and data extraction will be performed independently and in duplicate. In Study I, we will assess methods used to evaluate HTE, and in Study II, we will assess adaptations and stopping rules in the included trials.

**Discussion:**

The two proposed methodological studies will provide an overview of important methodological features regarding the assessment of HTE and adaptations used in adaptive platform trials. Better knowledge of available methods to assess these features can improve the conditions for designing adaptive platform trials and identify areas for further development.

## 1 Background

In adaptive trials, results from planned adaptive (interim) analyses can modify the ongoing trial according to pre‐specified rules that preserve trial integrity and validity [1]. Recently, more advanced adaptive trial designs (with more adaptive analyses and multiple types of adaptations) have received much attention, and adaptive platform trials increasingly emerge [2]. These trials enable assessment of multiple interventions in a specific population, with the addition of new interventions to the platform in a potentially perpetual manner [3, 4, 5, 6]. Additionally, adaptive platform trials can alleviate challenges of conventional randomised clinical trials (RCTs), such as lack of flexibility and the frequent ‘inconclusive’ trial results due to over‐optimistic anticipated effect sizes and incorrect assumptions in sample size estimations [5, 7].

A recent methodological study summarised features of adaptive platform trials with publications up to 2022 [2]. Among 98 unique platform trials, 33% were in cancer and 38% were in *Covid‐19* populations, most studied drugs‐related interventions (88%), and most were open‐label (72%). More than half of the trials (66%) used frequentist statistical methods and fixed randomisation (59%), although Bayesian statistical methods and response‐adaptive randomisation were used in 29% and 32%, respectively [2]. In addition to these general design features, several other considerations are relevant for adaptive platform trials. These include how heterogeneity of treatment effects (HTE) is assessed, and the design of adaptive analyses and corresponding adaptation rules, including those for adaptive stopping.

### 1.1 Heterogeneity of Treatment Effects

When a treatment effect differs according to patient characteristics, it is referred to as HTE (or heterogeneity in intervention effects, HIE) [8]. In medical research, broad access to research participation is encouraged simultaneously with the overall aim of detecting average treatment effects, although there is often hope for individualised treatment strategies [8, 9, 10]. Thus, especially for broad and potentially heterogeneous populations, the rationale for studying HTE appears clear. It may be assessed in conventional subgroup analyses (“1‐variable‐at‐a‐time”‐type analyses), but these are often hampered by low power, dichotomisation/categorisation, multiplicity issues, and potential additivity of effects or effect modification by other variables [11, 12]. More advanced assessments of HTE include predictive approaches that consider multiple baseline characteristics simultaneously in prediction models of outcomes, or by use of machine learning algorithms [8, 11, 13], although several of these techniques require more participants than conventional methods [14].

Because adaptive platform trials generally are designed to have higher probabilities of generating firm evidence on patient outcomes compared with conventional trials, they may offer better opportunities to assess HTE [3]. Additionally, in advanced adaptive designs, the ongoing trial may be modified according to identified HTE (e.g., by stratum‐specific adaptations or enrichment of the population) and in those cases, the advantage of any HTE identification can be harnessed while the trial is ongoing and not purely limited to after trial completion [1, 5].

### 1.2 Adaptations

Recruitment to interventions within adaptive platform trials may be stopped, for example, for superiority/inferiority (i.e., benefit/harm), equivalence, futility, or non‐inferiority before a maximal sample size (or be designed without a maximum sample size at all [3, 15]), to lower the necessary sample size to detect clinically important differences—or the lack thereof—in outcomes [4, 5]. In multi‐arm (> 2 arm) trials/domains, arms may also be dropped early for similar reasons [4, 5]. Other adaptive features can include response‐adaptive randomisation, where allocation probabilities are updated based on adaptive analyses of outcome data, that is, allocation is increased to arms with higher probabilities of being superior [4], or population enrichment, where inclusion criteria are restricted to those more likely to benefit [4, 16]. Moreover, trials/domains may employ adaptations (including stopping, arm‐dropping, and response‐adaptive randomisation) separately in specific strata, so‐called stratum‐specific adaptations [4]. All of these adaptations may be protocolised in multiple ways including with respect to the reasons for stopping, the format of the stopping rules, types of response‐adaptive randomisation, inclusion restrictions in use, and how frequently adaptations are possible. Further, platform trials may use some but not all adaptive features at the same time.

### 1.3 Aims

We expect that varying practices exist both with regards to HTE assessment and adaptive analyses (including adaptation rules), and that an overview of the methodology in existing platform trials may help trialists design future platform trials or domains [1], or update existing ones. It will also help identify areas, which may require further research. In two methodological studies outlined here, we aim to provide an overview of adaptive platform trials and summarise their methodological characteristics with a focus on the assessment of HTE and adaptations.

## 2 Methods

We report this protocol according to the Preferred Reporting Items for Systematic review and Meta‐Analysis Protocols (PRISMA‐P) statement and the guidelines by Murad and Wang for reporting meta‐epidemiological methodology research [17, 18]. Completed checklists are available in Supporting Information: Appendix 3–4. The present protocol will be published in a peer‐reviewed journal prior to data extraction.

The protocol outlines two methodological studies based on a common systematic literature search and review:

*Study I*. Assessment of heterogeneity of treatment effects in adaptive platform trials.

*Study II*. Use of adaptations including adaptive stopping in adaptive platform trials.

### 2.1 Search Strategy

We will conduct a systematic literature search to identify adaptive platform trials using a search strategy, which has been developed in collaboration with an experienced research librarian, building on that developed in a previous study [2]. The search is designed to identify adaptive platform trials according to our specified eligibility criteria and additionally employs the Cochrane Highly Sensitive Search Strategy for identifying randomised trials [19]. We will search MEDLINE, CENTRAL, ClinicalTrials.gov, and the EU Clinical Trials Register (EU CTR) and Clinical Trials Information System (CTIS) from January 2005 for protocols or results of platform trials. Additionally, we will review citations of previous relevant manuscripts of platform trials [2, 5]. The full search strategy for MEDLINE and additional details are available in Supporting Information: Appendix 1.

### 2.2 Eligibility

We will include platform trials based on the following inclusion criteria:

- Adaptive platform trials including multi‐arm multi‐stage (MAMS) trials [6] (in the following protocol text, both designs will be referred to as adaptive platform trials).
- Trials involving human participants.

We will include peer‐reviewed publications, and additionally include preprints, abstracts, and technical documents if identified online or by contact with trial investigators. We will include both primary publications of results and protocols from adaptive platform trials. We will make no language restrictions, or restrictions regarding population, interventions, comparator, or outcomes being studied. We will include all relevant documents for each trial (including protocols, registrations etc.) to be able to extract the predefined data of interest.

### 2.3 Screening and Selection

**FIGURE 1:**
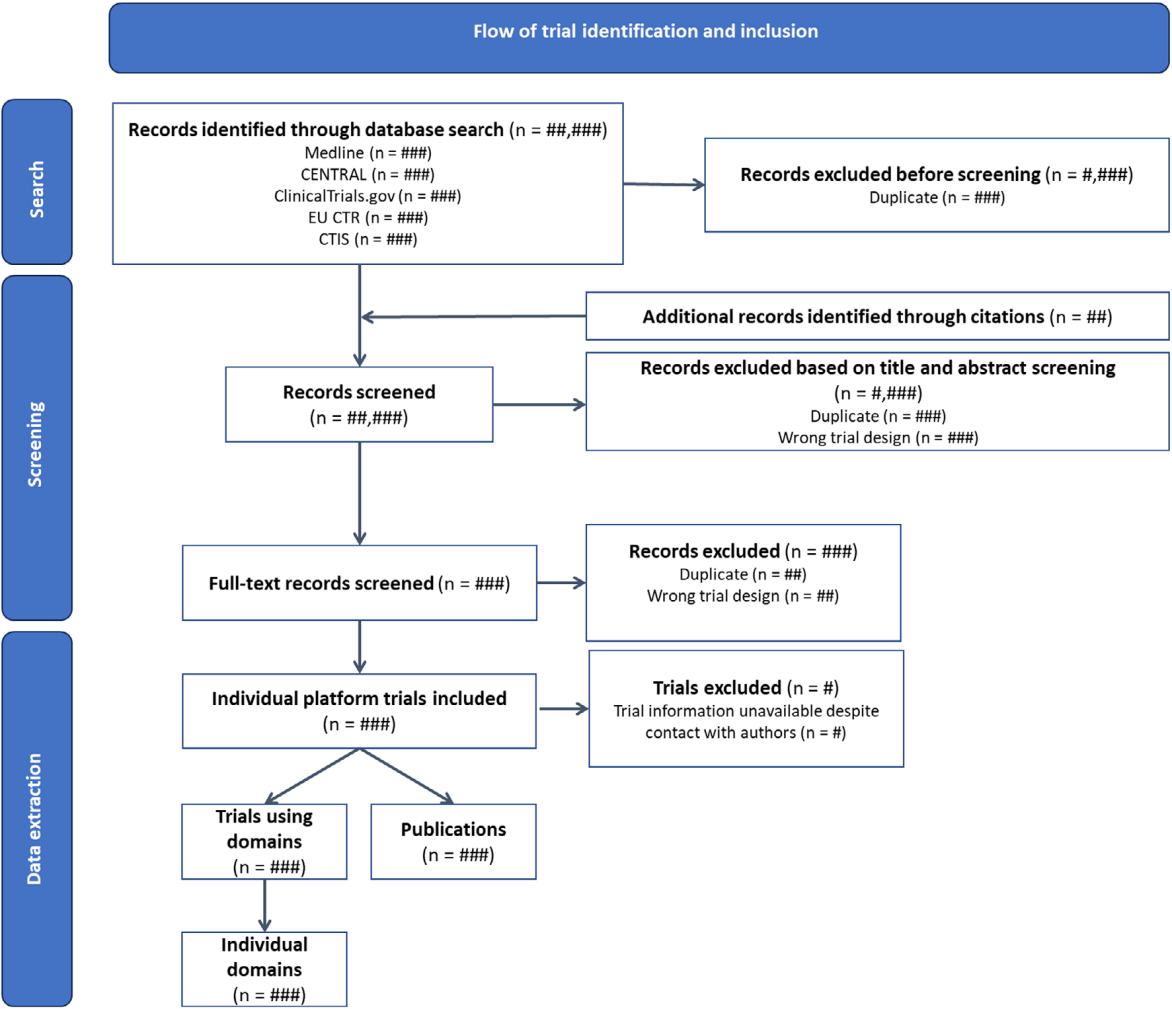
Screening and inclusion flowchart. [Applicable for both studies] mock figure. Mock screening and inclusion flowchart adapted from the PRISMA flowchart [20]. The flow of screening will be common for Studies I and II, including the final number of platform trials included. CTIS, Clinical Trials Information System; EU CTR, EU Clinical Trials Register.

A minimum of two reviewers working independently and in duplicate will screen titles, abstracts, and full‐text documents of retrieved records (Figure 1). Any discrepancies will be resolved in consensus with a third author. For any trial without a publicly available protocol, we will contact investigators to ask if they will share their protocol. We will use the Covidence systematic review software for screening (Veritas Health Innovation, Melbourne, Australia; www.covidence.org).

### 2.4 Data Sources, Extraction, and Synthesis

A minimum of two reviewers working independently and in duplicate will extract data from included trials. Prior to the initiation of the screening and data extraction process, we will develop a data‐extraction form including definitions of all data points, which will be pilot tested on 15–20 relevant adaptive platform trials and adjusted as necessary. The final data‐extraction forms will be available in the supplements to the final manuscripts of Studies I and II.

For both studies, we will extract data on individual trial characteristics (name/acronym, available documents, intervention types, recruitment status, year of first inclusion, most recent reporting of results, number of completed/active domains or completed/active arms, and registration numbers), and general methodological characteristics (population, intervention types, types of comparisons, types of primary outcomes, and statistical paradigm) as outlined in Tables 1 and S1.

**TABLE 1:** Overall characteristics in the included trials: [applicable for both studies] mock table.

Characteristic	All trials (*n* = ##)	Phase I–II drug trials (*n* = ##)	Phase III–IV drug trials (*n* = ##)	Other intervention type trials (*n* = ##)
	Number (%)			
Population				
Infectious diseases	## (##.#%)	## (##.#%)	## (##.#%)	## (##.#%)
Covid‐19	## (##.#%)	## (##.#%)	## (##.#%)	## (##.#%)
Pneumonia (excluding Covid‐19 only population)	## (##.#%)	## (##.#%)	## (##.#%)	## (##.#%)
Oncology/haematology	## (##.#%)	## (##.#%)	## (##.#%)	## (##.#%)
ICU/critical care	## (##.#%)	## (##.#%)	## (##.#%)	## (##.#%)
Primary care	## (##.#%)	## (##.#%)	## (##.#%)	## (##.#%)
Neurology	## (##.#%)	## (##.#%)	## (##.#%)	## (##.#%)
Surgical	## (##.#%)	## (##.#%)	## (##.#%)	## (##.#%)
Paediatric	## (##.#%)	## (##.#%)	## (##.#%)	## (##.#%)
Psychiatry	## (##.#%)	## (##.#%)	## (##.#%)	## (##.#%)
Intervention type^a^				
Drug	## (##.#%)	—	—	—
Phase I	## (##.#%)	—	—	—
Phase II	## (##.#%)	—	—	—
Phase III	## (##.#%)	—	—	—
Phase IV	## (##.#%)	—	—	—
Seamless coverage of several drug phases	## (##.#%)	—	—	—
Management	## (##.#%)	—	—	—
Medical device	## (##.#%)	—	—	—
Surgical/procedural	## (##.#%)	—	—	—
Mixed	## (##.#%)	—	—	—
Types of comparisons^b^				
Pairwise comparisons against a common control group	## (##.#%)	## (##.#%)	## (##.#%)	## (##.#%)
Simultaneous “all‐versus‐all” comparisons	## (##.#%)	## (##.#%)	## (##.#%)	## (##.#%)
Mixed	## (##.#%)	## (##.#%)	## (##.#%)	## (##.#%)
Types of primary outcomes^c^				
Mortality	## (##.#%)	## (##.#%)	## (##.#%)	## (##.#%)
“Days alive without X” type	## (##.#%)	## (##.#%)	## (##.#%)	## (##.#%)
Morbidity type^d^	## (##.#%)	## (##.#%)	## (##.#%)	## (##.#%)
Functional/health‐related quality of life‐type^e^	## (##.#%)	## (##.#%)	## (##.#%)	## (##.#%)
Service‐related/surrogate^f^	## (##.#%)	## (##.#%)	## (##.#%)	## (##.#%)
Statistical paradigm				
Frequentist	## (##.#%)	## (##.#%)	## (##.#%)	## (##.#%)
Bayesian	## (##.#%)	## (##.#%)	## (##.#%)	## (##.#%)
Mixed^g^	## (##.#%)	## (##.#%)	## (##.#%)	## (##.#%)
Mixed at domain level	## (##.#%)	## (##.#%)	## (##.#%)	## (##.#%)
Mixed at platform level	## (##.#%)	## (##.#%)	## (##.#%)	## (##.#%)

*Note:* Summary of methodological characteristics in the included trials stratified by phases of drug trials and other intervention type trials, numbers (percentages). For several characteristics, the included trials may appear in several categories, and the percentages therefore may not add up to 100%. Other distinct categories discovered during data extraction will be added or categories removed as deemed relevant. Minor categories for each characteristic will be collected in common categories labelled “other” (and detailed in Supporting Information to the final manuscript). If some data points are unobtainable for specific trials, we will add a category labelled ‘not reported/unclear’.

Abbreviations: Covid‐19, coronavirus disease 2019; ICU, intensive care unit.

^a^ We will report intervention types in categories inspired by previous reporting in critical care studies [21, 22]. Drug interventions will be categorised according to phases (phase I, II, III, or IV or seamless coverage of several drug phases); these will also be specified for the individual trials as outlined in Table S1.

^b^ We will report types of comparisons at domain‐level. With a common control arm, all other arms are compared pairwise against it with adaptive decisions based on probabilities from the pairwise comparisons. With “all‐versus‐all” comparisons there is no common control arm, and adaptive decisions are based on the probabilities of each arm being overall best or all arms being practically equivalent [3, 4].

^c^ We will report the use of all outcomes labelled as primary outcome or guiding outcome, that is, the outcome used to drive adaptations, which is most commonly the same as the primary outcome. As some trials will use domains, the total number of primary outcomes will not add up to the number of included trials. If a trial uses just one primary outcome across all domains, we will count the outcome just once at the trial level.

^d^ Morbidity‐type outcomes will include outcomes related to adverse events/reactions and similar patient‐important outcomes.

^e^ Functional‐type outcomes will include scorings of, for example, health‐related quality‐of‐life, cognitive/neurological function, or similar patient‐important outcome scales.

^f^ Service‐related/surrogate‐type outcomes will include outcomes related to care (i.e., economy, climate or equipment related outcomes) or non‐patient important outcomes (i.e., blood pressure or biomarkers).

^g^ Mixed at domain level means that one domain uses both frequentist and Bayesian statistics, whereas mixed at platform level can mean that different domains on the platform use statistical paradigms.

For Study I, we will additionally extract data on HTE analysis features as outlined in Tables 2 and 3 (Table 2 includes data on the general characteristics of HTE assessment and Table 3 includes data on the methods used to assess HTE).

**TABLE 2:** Characteristics of heterogeneity of treatment effect (HTE) assessment in the included trials: [Study I, HTE] mock table.

Characteristic	All trials (*n* = ##)	Phase I–II drug trials (*n* = ##)	Phase III–IV drug trials (*n* = ##)	Other intervention type trials (*n* = ##)
	Number (%) or median (IQR) [range]			
Protocolisation of HTE assessment—number (%)				
Pre‐planned	## (##.#%)	## (##.#%)	## (##.#%)	## (##.#%)
Post hoc	## (##.#%)	## (##.#%)	## (##.#%)	## (##.#%)
HTE not assessed	## (##.#%)	## (##.#%)	## (##.#%)	## (##.#%)
Timing of HTE assessment—number (%)				
After conclusion of participant enrolment and follow‐up	## (##.#%)	## (##.#%)	## (##.#%)	## (##.#%)
During participant enrolment	## (##.#%)	## (##.#%)	## (##.#%)	## (##.#%)
Baseline variables assessed for HTE^a^—number (%)				
Comorbidities	## (##.#%)	## (##.#%)	## (##.#%)	## (##.#%)
Sub‐category of disease	## (##.#%)	## (##.#%)	## (##.#%)	## (##.#%)
Severity of illness	## (##.#%)	## (##.#%)	## (##.#%)	## (##.#%)
Outcome prediction model^b^	## (##.#%)	## (##.#%)	## (##.#%)	## (##.#%)
Biomarkers	## (##.#%)	## (##.#%)	## (##.#%)	## (##.#%)
Received interventions	## (##.#%)	## (##.#%)	## (##.#%)	## (##.#%)
Use of devices	## (##.#%)	## (##.#%)	## (##.#%)	## (##.#%)
Setting‐based population^c^	## (##.#%)	## (##.#%)	## (##.#%)	## (##.#%)
Other	## (##.#%)	## (##.#%)	## (##.#%)	## (##.#%)
Variable‐types assessed for HTE^a^—number (%)				
Dichotomous	## (##.#%)	## (##.#%)	## (##.#%)	## (##.#%)
Ordinal	## (##.#%)	## (##.#%)	## (##.#%)	## (##.#%)
Nominal	## (##.#%)	## (##.#%)	## (##.#%)	## (##.#%)
Continuous/numeric	## (##.#%)	## (##.#%)	## (##.#%)	## (##.#%)
Categorised numeric variable	## (##.#%)	## (##.#%)	## (##.#%)	## (##.#%)
Dichotomised numeric or categorical variable	## (##.#%)	## (##.#%)	## (##.#%)	## (##.#%)
Other characteristics				
Number of variables assessed for HTE—median (IQR) [range]	# (# to #) [# to #]	# (# to #) [# to #]	# (# to #) [# to #]	# (# to #) [# to #]
HTE assessed for non‐primary outcome—number (%)	## (##.#%)	## (##.#%)	## (##.#%)	## (##.#%)
Stakeholder involvement^d^—number (%)	## (##.#%)	## (##.#%)	## (##.#%)	## (##.#%)

*Note:* Summary of overall characteristics of HTE assessment in the included trials stratified by phases of drug trials and other intervention type trials, numbers (percentages), medians (IQRs), and [full ranges]. For several characteristics, the included trials/domains may appear in several categories (e.g., due to different domains on the platform using different methods), and the percentages therefore may not add up to 100%. Other distinct categories discovered during data extraction will be added or categories removed as deemed relevant. Minor categories for each characteristic will be collected in common categories labelled “other” (and detailed in Supporting Information to the final manuscript). If some data points are unobtainable for specific trials, we will add a category labelled “not reported/unclear.”

Abbreviations: HTE, heterogeneity of treatment effects; IQR, interquartile range.

^a^ We will only include analyses of pre‐randomization/baseline variables.

^b^ Use of a complete/previously developed data model for outcome prediction.

^c^ Relevant for trials/domains enrolling patients across multiple settings, for example, prehospital and emergency department.

^d^ If stakeholder involvement in HTE assessment is not explicitly stated in the retrieved documents, we will assume that stakeholders were not involved.

**TABLE 3:** Methods used to assess heterogeneity of treatment effect (HTE) in the included trials (Study I, HTE) mock table.

Characteristic	All trials (*n* = ##)	Phase I–II drug trials (*n* = ##)	Phase III–IV drug trials (*n* = ##)	Other intervention type trials (*n* = ##)
	Number (%)			
Analysis method for HTE				
Conventional subgroup analysis (“1 variable at a time” approach)	## (##.#%)	## (##.#%)	## (##.#%)	## (##.#%)
Risk‐modelling approach^a^	## (##.#%)	## (##.#%)	## (##.#%)	## (##.#%)
Internally developed risk model	## (##.#%)	## (##.#%)	## (##.#%)	## (##.#%)
Externally developed risk model	## (##.#%)	## (##.#%)	## (##.#%)	## (##.#%)
Primary outcome included in model	## (##.#%)	## (##.#%)	## (##.#%)	## (##.#%)
Primary outcome not included in model	## (##.#%)	## (##.#%)	## (##.#%)	## (##.#%)
Effect modelling approach^b^	## (##.#%)	## (##.#%)	## (##.#%)	## (##.#%)
Risk magnification approach^c^	## (##.#%)	## (##.#%)	## (##.#%)	## (##.#%)
Machine learning/AI‐derived model^d^	## (##.#%)	## (##.#%)	## (##.#%)	## (##.#%)
Supervised	## (##.#%)	## (##.#%)	## (##.#%)	## (##.#%)
Unsupervised	## (##.#%)	## (##.#%)	## (##.#%)	## (##.#%)
Semi‐supervised	## (##.#%)	## (##.#%)	## (##.#%)	## (##.#%)
Self‐supervised	## (##.#%)	## (##.#%)	## (##.#%)	## (##.#%)
Reinforcement learning	## (##.#%)	## (##.#%)	## (##.#%)	## (##.#%)
Hierarchical models	## (##.#%)	## (##.#%)	## (##.#%)	## (##.#%)
Statistical paradigm used				
Frequentist	## (##.#%)	## (##.#%)	## (##.#%)	## (##.#%)
Bayesian	## (##.#%)	## (##.#%)	## (##.#%)	## (##.#%)
Mixed	## (##.#%)	## (##.#%)	## (##.#%)	## (##.#%)
Type of prior(s) for HTE in Bayesian analyses				
Neutral	## (##.#%)	## (##.#%)	## (##.#%)	## (##.#%)
Opinion‐based^e^	## (##.#%)	## (##.#%)	## (##.#%)	## (##.#%)
Evidence‐based	## (##.#%)	## (##.#%)	## (##.#%)	## (##.#%)
Strength of prior(s) for HTE in Bayesian analyses				
Uninformative	## (##.#%)	## (##.#%)	## (##.#%)	## (##.#%)
Weakly informative	## (##.#%)	## (##.#%)	## (##.#%)	## (##.#%)
Moderately to strongly informative	## (##.#%)	## (##.#%)	## (##.#%)	## (##.#%)
A priori hypothesised direction expected treatment effect				
Yes	## (##.#%)	## (##.#%)	## (##.#%)	## (##.#%)
Planned adaptations according to HTE^f^				
Population enrichment	## (##.#%)	## (##.#%)	## (##.#%)	## (##.#%)
Separate response‐adaptive randomisation in strata	## (##.#%)	## (##.#%)	## (##.#%)	## (##.#%)
Stopping/arm‐dropping in strata	## (##.#%)	## (##.#%)	## (##.#%)	## (##.#%)
No adaptations according to HTE	## (##.#%)	## (##.#%)	## (##.#%)	## (##.#%)

*Note:* Summary of overall analysis methods for HTE in the included trials stratified by phases of drug trials and other intervention type trials, numbers (percentages). For several characteristics, the included trials/domains may appear in several categories (e.g., due to different domains on the platform using different methods), and the percentages therefore may not add up to 100%. Other distinct categories discovered during data extraction will be added or categories removed as deemed relevant. Minor categories for each characteristic will be collected in common categories labelled “other” (and detailed in Supporting Information to the final manuscript). If some data points are unobtainable for specific trials, we will add a category labelled “not reported/unclear.”

Abbreviations: AI, artificial intelligence; HTE, heterogeneity of treatment effects; RCT, randomised clinical trial.

^a^ Risk modelling refers to analyses where a multivariable model that predicts the risk of an outcome (usually the primary outcome) is used to examine risk‐based variation in treatment effects [23].

^b^ Effect modelling refers to analyses where a model is developed on RCT data with inclusion of treatment assignment variables and potential inclusion of treatment interaction terms, for example, in a regression model [23].

^c^ Risk magnification refers to analyses where “high‐risk patients” are identified without any data‐based assumption of a relative treatment effect [23, 24].

^d^ We plan to extract exact descriptions of machine learning/AI‐derived models and report the exact descriptions alongside the grouping in the overall categories outlined in this mock table.

^e^ In favour of one intervention (not evidence‐based) including expert‐elicited priors.

^f^ Only relevant for trials with HTE assessment during recruitment as planned to be reported in Table 2 (mock table).

For Study II, we will additionally extract data on adaptations and stopping rules as outlined in Tables 4 and 5 (Table 4 includes data on planning of adaptations and stopping and Table 5 includes data on results of adaptations and stopping in trials where arms/domains have completed such analyses).

**TABLE 4:** Planning of adaptations and stopping in the included trials: [Study II, adaptations and stopping] mock table.

Characteristic	All trials (*n* = ##)	Phase I–II drug trials (*n* = ##)	Phase III–IV drug trials (*n* = ##)	Other intervention type trials (*n* = ##)
	Number (%) or median (IQR) [range]			
Maximum sample size specified—number (%)				
Yes	## (##.#%)	## (##.#%)	## (##.#%)	## (##.#%)
Maximum sample size—median (IQR) [range]	# (# to ##) [# to #]	# (# to ##) [# to #]	# (# to ##) [# to #]	# (# to ##) [# to #]
No	## (##.#%)	## (##.#%)	## (##.#%)	## (##.#%)
Range of expected sample sizes under different scenarios—median (IQR) [range]				
Minimum sample size	# (# to ##) [# to #]	# (# to ##) [# to #]	# (# to ##) [# to #]	# (# to ##) [# to #]
Maximum sample size	# (# to ##) [# to #]	# (# to ##) [# to #]	# (# to ##) [# to #]	# (# to ##) [# to #]
Triggers of adaptive analyses—number (%)				
Fixed time‐points	## (##.#%)	## (##.#%)	## (##.#%)	## (##.#%)
Number of participants	## (##.#%)	## (##.#%)	## (##.#%)	## (##.#%)
Number of events	## (##.#%)	## (##.#%)	## (##.#%)	## (##.#%)
Maximum number of analyses specified—number (%)				
Yes	## (##.#%)	## (##.#%)	## (##.#%)	## (##.#%)
Total number—median (IQR) [range]	# (# to ##) [# to #]	# (# to ##) [# to #]	# (# to ##) [# to #]	# (# to ##) [# to #]
No	## (##.#%)	## (##.#%)	## (##.#%)	## (##.#%)
No upper limit	## (##.#%)	## (##.#%)	## (##.#%)	## (##.#%)
First adaptive analysis—median (IQR) [range]				
Percentage of max sample size/events required before first analysis	# (# to ##) [# to #]	# (# to ##) [# to #]	# (# to ##) [# to #]	# (# to ##) [# to #]
Adaptations planned according to adaptive analyses—number (%)				
Stopping	## (##.#%)	## (##.#%)	## (##.#%)	## (##.#%)
Reasons				
Superiority/inferiority	## (##.#%)	## (##.#%)	## (##.#%)	## (##.#%)
Equivalence	## (##.#%)	## (##.#%)	## (##.#%)	## (##.#%)
Futility	## (##.#%)	## (##.#%)	## (##.#%)	## (##.#%)
Non‐inferiority	## (##.#%)	## (##.#%)	## (##.#%)	## (##.#%)
Arm‐dropping (for trials/domains with > 2 arms)	## (##.#%)	## (##.#%)	## (##.#%)	## (##.#%)
Response‐adaptive randomisation	## (##.#%)	## (##.#%)	## (##.#%)	## (##.#%)
Restricted^a^	## (##.#%)	## (##.#%)	## (##.#%)	## (##.#%)
Unrestricted	## (##.#%)	## (##.#%)	## (##.#%)	## (##.#%)
Population enrichment	## (##.#%)	## (##.#%)	## (##.#%)	## (##.#%)
Stratum‐specific adaptations Stopping Arm‐dropping (for trials/domains with > 2 arms) Response‐adaptive randomisation Restricted^a^ Unrestricted	## (##.#%) ## (##.#%) ## (##.#%) ## (##.#%) ## (##.#%) ## (##.#%) ## (##.#%)	## (##.#%) ## (##.#%) ## (##.#%) ## (##.#%) ## (##.#%) ## (##.#%) ## (##.#%)	## (##.#%) ## (##.#%) ## (##.#%) ## (##.#%) ## (##.#%) ## (##.#%) ## (##.#%)	## (##.#%) ## (##.#%) ## (##.#%) ## (##.#%) ## (##.#%) ## (##.#%) ## (##.#%)
Shapes of stopping boundaries^b^—number (%)				
Constant (Pocock‐like)	## (##.#%)	## (##.#%)	## (##.#%)	## (##.#%)
Decreasingly strict (O'Brien‐Fleming‐like)	## (##.#%)	## (##.#%)	## (##.#%)	## (##.#%)
Strict until last (Haybittle‐Peto‐like)	## (##.#%)	## (##.#%)	## (##.#%)	## (##.#%)
Planned consequences of crossing stopping boundaries^c^—number (%)				
Binding stopping rules	## (##.#%)	## (##.#%)	## (##.#%)	## (##.#%)
Non‐binding stopping rules	## (##.#%)	## (##.#%)	## (##.#%)	## (##.#%)
Mixed	## (##.#%)	## (##.#%)	## (##.#%)	## (##.#%)
Simulation and calibration of stopping rules^d^—number (%)				
Simulation based evaluation of type 1 error rates	## (##.#%)	## (##.#%)	## (##.#%)	## (##.#%)
Simulation based calibration of stopping rules to target desired type 1 error rate	## (##.#%)	## (##.#%)	## (##.#%)	## (##.#%)
No assessment of type 1 error rates	## (##.#%)	## (##.#%)	## (##.#%)	## (##.#%)
Guiding outcome for adaptive analyses—number (%)				
Primary outcome	## (##.#%)	## (##.#%)	## (##.#%)	## (##.#%)
Non‐primary outcome	## (##.#%)	## (##.#%)	## (##.#%)	## (##.#%)
Same as primary outcome but after different follow‐up duration	## (##.#%)	## (##.#%)	## (##.#%)	## (##.#%)
Completely different outcome	## (##.#%)	## (##.#%)	## (##.#%)	## (##.#%)
Type of prior(s) for treatment effect(s) in Bayesian adaptive analyses—number (%)				
Neutral	## (##.#%)	## (##.#%)	## (##.#%)	## (##.#%)
Opinion‐based prior^e^	## (##.#%)	## (##.#%)	## (##.#%)	## (##.#%)
Evidence‐based	## (##.#%)	## (##.#%)	## (##.#%)	## (##.#%)
Strength of prior(s) for treatment effect(s) in Bayesian adaptive analyses—number (%)				
Uninformative	## (##.#%)	## (##.#%)	## (##.#%)	## (##.#%)
Weakly informative	## (##.#%)	## (##.#%)	## (##.#%)	## (##.#%)
Moderately to strongly informative	## (##.#%)	## (##.#%)	## (##.#%)	## (##.#%)

*Note:* Summary of overall analysis methods for adaptations in the included trials stratified by phases of drug trials and other intervention type trials, numbers (percentages), medians (interquartile ranges), or ranges (minimum to maximum).For several characteristics, the included trials/domains may appear in several categories (e.g., due to different domains on the platform using different methods), and the percentages, therefore, may not add up to 100%. Other distinct categories discovered during data extraction will be added or categories removed as deemed relevant. Minor categories for each characteristic will be collected in common categories labelled “other” (and detailed in Supporting Information to the final manuscript). If some data points are unobtainable for specific trials, we will add a category labelled “not reported/unclear.”

Abbreviation: IQR, interquartile range.

^a^ Restricted response‐adaptive randomisation refers to predefined restrictions to avoid over‐aggressive adaptations to random fluctuations [3, 4].

^b^ Shape of stopping boundaries regardless of statistical paradigm used, categorised according to the form they mostly resemble of the three most typical forms of monitoring boundaries used in conventional group sequential designs [25].

^c^ Whether planned consequences of crossing stopping boundaries at adaptive analyses are binding (ultimate, i.e., they must be adhered to) or non‐binding, for example, involving suggestions by the data monitoring and safety board (DSMB).

^d^ Statistical simulation of trials to evaluate the performance of the trial design under various clinical scenarios (e.g., small/large benefit/harm of intervention) and assumptions, for example, various inclusion rates, various outcome distributions in reference population, and so forth [3]. These simulations may, in turn, be used to calibrate stopping rules, that is, to automatically update stopping rules to ensure a targeted type 1 error rate is achieved.

^e^ In favour of one intervention (not explicitly based on previous evidence) including expert‐elicited priors.

**TABLE 5:** Results of adaptations and stopping in the included trials: [Study II, adaptations and stopping] mock table.

Characteristic	All trials (*n* = ##)	Phase I–II drug trials (*n* = ##)	Phase III–IV drug trials (*n* = ##)	Other intervention type trials (*n* = ##)
	Number (%) or median (IQR) [range]			
Reason for adaptive stopping—number (%)				
Superiority/inferiority	## (##.#%)	## (##.#%)	## (##.#%)	## (##.#%)
Equivalence	## (##.#%)	## (##.#%)	## (##.#%)	## (##.#%)
Futility	## (##.#%)	## (##.#%)	## (##.#%)	## (##.#%)
Non‐inferiority	## (##.#%)	## (##.#%)	## (##.#%)	## (##.#%)
Included population—median (IQR) [range]				
Percentage of maximum sample size included	# (# to ##) [# to #]	# (# to ##) [# to #]	# (# to ##) [# to #]	# (# to ##) [# to #]
Other adaptations performed—number (%)				
Arm‐dropping (if > 2 arms)	## (##.#%)	## (##.#%)	## (##.#%)	## (##.#%)
Response‐adaptive randomisation^a^	## (##.#%)	## (##.#%)	## (##.#%)	## (##.#%)
Lowest allocation probability for any arm—median (IQR) [range]	# (# to #) [# to #]	# (# to #) [# to #]	# (# to #) [# to #]	# (# to #) [# to #]
Highest allocation probability any arm—median (IQR) [range]	# (# to #) [# to #]	# (# to #) [# to #]	# (# to #) [# to #]	# (# to #) [# to #]
Population enrichment	## (##.#%)	## (##.#%)	## (##.#%)	## (##.#%)
Stratum‐specific adaptations	## (##.#%)	## (##.#%)	## (##.#%)	## (##.#%)
Stopping	## (##.#%)	## (##.#%)	## (##.#%)	## (##.#%)
Arm‐dropping (if > 2 arms)	## (##.#%)	## (##.#%)	## (##.#%)	## (##.#%)
Response‐adaptive randomisation	## (##.#%)	## (##.#%)	## (##.#%)	## (##.#%)
Stopping for other than pre‐specified reason—number (%)				
DSMB recommendation for other reason than stopping rule	## (##.#%)	## (##.#%)	## (##.#%)	## (##.#%)
Other reason	## (##.#%)	## (##.#%)	## (##.#%)	## (##.#%)

*Note:* Summary of overall results of adaptive analyses and stopping in the included trials/domains stratified by phases of drug trials and other intervention type trials, only relevant for trials with publications. Numbers (percentages), medians (interquartile ranges) or ranges (minimum to maximum). For several characteristics, the included trials/domains may appear in several categories (e.g., due to different domains on the platform using different methods), and the percentages therefore may not add up to 100%. Other distinct categories discovered during data extraction may be added or categories may be removed as deemed relevant. Minor categories for each characteristic will be collected in common categories labelled ‘other’ (and detailed in Supporting Information to the final manuscript). If some data points are unobtainable for specific trials, we will add a category labelled ‘not reported/unclear’.

Abbreviations: DSMB, Data Safety and Monitoring Board; IQR, interquartile range.

^a^ For results of response‐adaptive randomisations, we will report the median (IQR) and [full range] of the lowest and highest allocation probabilities in percentages, respectively.

The study is methodologically focused, and we will not assess data on patient outcomes. We will iteratively review the data extraction forms and will add, modify, or remove categories from the planned mock tables as deemed relevant during data extraction (the rationale for any changes will be documented and reported in the final manuscripts).

### 2.5 Outcomes and Data Synthesis

Results will be summarised using descriptive statistics, that is, numbers and percentages and medians with interquartile and full ranges. Outcomes of interest are methodological characteristics of the included trials as outlined above. For both studies, we will stratify the extracted data by phases of drug trials (phase I–II/phase III–IV [26, 27]) and other intervention type trials, respectively, as we expect that methodological choices may substantially vary according to this. Seamless platform trials covering several phases will be classified according to the latest phase, unless clearly stated otherwise with reasoning.

### 2.6 Ethics

Ethics approval is not required as we will only summarise clinical trials and not use any participant data.

### 2.7 Reporting

We will publish the results of Studies I and II in separate publications in international, peer‐reviewed scientific journal(s). We will duly report and justify any deviations from this protocol in both publications.

## 3 Discussion

The two proposed methodological studies will summarise methodological features of HTE assessment and adaptations in adaptive platform trials registered or published within the past 20 years. We expect that multiple features will be in use with regard to the design of these analyses and, to our knowledge, their use and characteristics have not previously been documented among adaptive platform trials.

Generally, limited knowledge of advanced adaptive trials, including adaptive platform trials, may result in suboptimal trial design and therefore continued oversight and evaluation of methods used in these trials will help researchers improve their understanding and design of trials. Ultimately, increased familiarity with advanced adaptive trial methodology may lead to improved adoption of these trials in medical communities, and better formal assessment and comparison of different methodological choices.

### 3.1 Strengths and Limitations

The proposed studies are strengthened through international collaboration, including input from clinicians, trialists, and statisticians to this common protocol. Our planned literature search expands the search period compared with a previous review of platform trials to potentially identify more relevant trials [2]. Further, we will include all documents containing relevant data regarding trial design and methodology, including through direct contact with trialists as necessary. Limitations of the studies include a risk that we may not be able to extract all relevant data points for some trials if these are identified at an early stage of protocolisation/registration. Especially for Study I, some HTE assessments of platform trials may be performed in secondary publications, and we cannot be sure to retrieve all secondary publications through our search strategy. We have not planned for continuous updates of the literature search and review in general, and results will thus inherently reflect the time of the search.

### 3.2 Perspectives

Perspectives of these methodological studies include improved knowledge of available methods for HTE assessment and adaptations in adaptive platform trials, and thereby improved conditions to assess the better strategies for these analyses in the future, both in the design of trials and in the development of guidelines for adaptive platform trial planning and conduct.

### 3.3 Conclusions

The two proposed methodological studies will provide an overview of adaptive platform trials with regard to their assessment of heterogeneity of treatment effects and adaptations and stopping.

## Author Contributions

Conceptualisation: T.S.M., A.K.G.J., AP, MHM, A.G. Methodology: all authors. Supervision: M.H.M., A.P., A.G. Visualisation: T.S.M., A.G. Writing – original draft: T.S.M. Writing – review and editing: all authors.

## Conflicts of Interest

T.S.M., A.K.G.J., A.P., M.H.M., and A.G. are involved in the design and conduct of the adaptive *Intensive Care Platform Trial (INCEPT)* (www.incept.dk). *INCEPT* has received grants from the *Novo Nordisk Foundation* and *Sygeforsikringen* “*danmark*,” and additional support from *Savværksejer Jeppe Juhl og hustru Ovita Juhls Mindelegat*, *Grosserer Jakob Ehrenreich og Hustru Grete Ehrenreichs Fond*, and *Dagmar Marshalls Fond*. Specific domains on *INCEPT* have received grants from *Danmarks Frie Forskningsfond*. The Department of Intensive Care at Rigshospitalet has received grants from the *Novo Nordisk Foundation* for other projects. M.K.C. is Chair of the *UK NIHR*/*MRC Better Methods Better Research Funding Panel* and has received multiple grants for the conduct of clinical trials from the *UK NIHR* programme. The other authors declare no conflicts of interest.

## Supporting information


**Data S1.** Supporting Information.

## Data Availability

No participant data were analysed in this protocol manuscript and the outlined studies will only summariseclinical trials and not participant data. The extracted data will be included in the final publications.
