# Supplementary material for: Adaptations and Heterogeneity of Treatment Effects in Platform Trials—Protocol for Two Methodological Studies
Source: Acta Anaesthesiol Scand. 2025 May 7;69(6):e70044. doi: 10.1111/aas.70044 (PMC12056685; doi:10.1111/aas.70044)
Supplement: Supplementary file 1 — Data S1. Supporting Information. [file AAS-69-0-s001.pdf]

Supplementary Appendix for

Adaptations and heterogeneity of treatment effects in platform trials – protocol for two methodological studies

Tine Sylvest Meyhoff[1], Aksel Karl Georg Jensen[1,2], Anders Perner[1,3], Ewan C. Goligher[4], Marion K. Campbell[5], Morten Hylander Møller[1,3], Anders Granholm[1,2]

[1] Department of Intensive Care, Copenhagen University Hospital – Rigshospitalet, Copenhagen, Denmark.

[2] Section of Biostatistics, Department of Public Health, University of Copenhagen, Copenhagen, Denmark.

[3] Department of Clinical Medicine, University of Copenhagen.

[4] Interdepartmental Division of Critical Care Medicine, University of Toronto, Toronto, Canada.

[5] Aberdeen Centre for Evaluation, University of Aberdeen, Aberdeen, United Kingdom.

TABLE OF CONTENTS

1. SEARCH STRATEGY 2
2. SUPPLEMENTARY TABLE 1. CHARACTERISTICS OF THE INCLUDED TRIALS 3
3. COMPLETED PRISMA-P REPORTING CHECKLIST 4
4. COMPLETED METHODOLOGY RESEARCH REPORTING CHECKLIST 6
5. REFERENCES 8

1. SEARCH STRATEGY

The search strategy below has been developed in collaboration with an experienced research librarian (Trine Lacoppidan Kæstel, information specialist and research librarian at Copenhagen University Hospital - Rigshospitalet, Copenhagen, Denmark), building on that developed in a previous study.[1] It is designed to identify adaptive platform trials according to our specified eligibility criteria, and additionally employs the Cochrane Highly Sensitive Search Strategy for identifying randomised trials.[2] We pilot-tested the search to secure identification of the platform trials included in a previous article from *the Adaptive Platform Trials Coalition*.[3] Any updates to the search will be reported with reasoning in the appendices to the final manuscripts. Search strategies including the dates of search for each database will also be reported in the final manuscripts appendices as shown below.
The full search strings for the remaining databases including dates of pilot and final searches will also be available in the Supplements to the final manuscripts of Studies I and II.

Search strategy, Medline. Pilot search: DD-MM-YYYY. Final search: DD-MM-YYYY.

Adaptive Clinical Trials as Topic/
exp Adaptive Clinical Trial/
((master or platform) adj3 protocol*).ti,ab,kf.
(platform* adj3 (study or studies or trial* or design*)).ab,ti,kf,pt.
((multi-arm or multiarm or multi-stage or multistage or mams or response-adaptive or adaptiv*) adj5 (design* or trial* or platform* or randomi#ed or randomi#ation)).ab,ti,kf,pt.
or/1-5
randomized controlled trial.pt.
controlled clinical trial.pt.
randomi#ed.ti,ab.
placebo*.ti,ab.
drug therapy.sh.
randomly.ti,ab.
trial.ti,ab.
groups.ti,ab.
exp animals/ not humans.sh.
or/7-14
16 not 15
6 and 17
limit 18 to yr="2005 -Current"

2. SUPPLEMENTARY TABLE 1. CHARACTERISTICS OF THE INCLUDED TRIALS [Applicable for both studies] Mock table

Name and acronym	Available documents (registration/protocols/ publications)	Intervention type[1]	Recruitment status (recruiting/not recruiting)	Year of first inclusion	Most recent reporting of results	Number of completed/ active domains	Number of completed/active arms	Registration numbers

Summary of supplementary characteristics in the included trials. This mock table will be included as a supplementary table in the final manuscripts of both studies. Other distinct categories discovered during data extraction will be added or categories removed as deemed relevant. If some data points are unobtainable for specific trials, we will report them as 'not reported/unclear'.

[1] We will extract data on intervention types at trial level categorised as specified for Table 1 in the main manuscript, i.e., drug (phase I, II, III or IV or seamless trial covering several phases including which phases), management, medical device, surgical/procedural or mixed trials.

3. COMPLETED PRISMA-P REPORTING CHECKLIST

PRISMA-P (Preferred Reporting Items for Systematic review and Meta-Analysis Protocols) 2015 checklist: recommended items to address in a systematic review protocol* [4]

Section and topic	Item No	Checklist item	Page No
**ADMINISTRATIVE INFORMATION**			
Title:			
Identification	1a	Identify the report as a protocol of a systematic review	1
Update	1b	If the protocol is for an update of a previous systematic review, identify as such	-
Registration	2	If registered, provide the name of the registry (such as PROSPERO) and registration number	-
Authors:			
Contact	3a	Provide name, institutional affiliation, e-mail address of all protocol authors; provide physical mailing address of corresponding author	1
Contributions	3b	Describe contributions of protocol authors and identify the guarantor of the review	1,8
Amendments	4	If the protocol represents an amendment of a previously completed or published protocol, identify as such and list changes; otherwise, state plan for documenting important protocol amendments	7
Support:			
Sources	5a	Indicate sources of financial or other support for the review	8
Sponsor	5b	Provide name for the review funder and/or sponsor	-
Role of sponsor or funder	5c	Describe roles of funder(s), sponsor(s), and/or institution(s), if any, in developing the protocol	-
**INTRODUCTION**			
Rationale	6	Describe the rationale for the review in the context of what is already known	3
Objectives	7	Provide an explicit statement of the question(s) the review will address with reference to participants, interventions, comparators, and outcomes (PICO)	4
**METHODS**			
Eligibility criteria	8	Specify the study characteristics (such as PICO, study design, setting, time frame) and report characteristics (such as years considered, language, publication status) to be used as criteria for eligibility for the review	4,5

Information sources	9	Describe all intended information sources (such as electronic databases, contact with study authors, trial registers or other grey literature sources) with planned dates of coverage	5
Search strategy	10	Present draft of search strategy to be used for at least one electronic database, including planned limits, such that it could be repeated	Supplementary Appendix p 2
Study records:			
Data management	11a	Describe the mechanism(s) that will be used to manage records and data throughout the review	5,6
Selection process	11b	State the process that will be used for selecting studies (such as two independent reviewers) through each phase of the review (that is, screening, eligibility and inclusion in meta-analysis)	5
Data collection process	11c	Describe planned method of extracting data from reports (such as piloting forms, done independently, in duplicate), any processes for obtaining and confirming data from investigators	5,6
Data items	12	List and define all variables for which data will be sought (such as PICO items, funding sources), any pre-planned data assumptions and simplifications	6
Outcomes and prioritization	13	List and define all outcomes for which data will be sought, including prioritization of main and additional outcomes, with rationale	6
Risk of bias in individual studies	14	Describe anticipated methods for assessing risk of bias of individual studies, including whether this will be done at the outcome or study level, or both; state how this information will be used in data synthesis	-
Data synthesis	15a	Describe criteria under which study data will be quantitatively synthesised	-
	15b	If data are appropriate for quantitative synthesis, describe planned summary measures, methods of handling data and methods of combining data from studies, including any planned exploration of consistency (such as I^2, Kendall's τ)	-
	15c	Describe any proposed additional analyses (such as sensitivity or subgroup analyses, meta-regression)	-
	15d	If quantitative synthesis is not appropriate, describe the type of summary planned	6, Mock tables 1-5 and Supplementary Table 1
Meta-bias(es)	16	Specify any planned assessment of meta-bias(es) (such as publication bias across studies, selective reporting within studies)	-
Confidence in cumulative evidence	17	Describe how the strength of the body of evidence will be assessed (such as GRADE)	-

From: Shamseer L, Moher D, Clarke M, Ghersi D, Liberati A, Petticrew M, Shekelle P, Stewart L, PRISMA-P Group. Preferred reporting items for systematic review and meta-analysis protocols (PRISMA-P) 2015: elaboration and explanation. BMJ. 2015 Jan 2;349(jan02 1):g7647.

4. COMPLETED METHODOLOGY RESEARCH REPORTING CHECKLIST

Proposed items to be used for reporting methodology research,[5] adapted from the PRISMA Checklist

Section and topic	Proposed item to be used in methodology research	Page No
**Title**		
Title	Identify the report as a meta-epidemiologic study	1
**Abstract**		2
Structured summary	Provide a structured summary that includes the background of the topic, goal of the study, data sources, method of data selection, appraisal and synthesis methods, results, limitations, conclusions and implications of key findings	
**Introduction**		3,4
Rationale	Describe the rationale for the meta-epidemiological study in the context of what is already known	
Objectives	Provide an explicit statement of the goal of the meta-epidemiological study and the hypothesis being empirically tested	
**Methods**		
Protocol	Indicate if a protocol exists, if and where it can be accessed (eg, Web address). Registration of a protocol is not mandatory	-
Eligibility criteria	Specify study characteristics used as criteria for eligibility with a rationale	5
Information sources	Describe all information sources (eg, databases with dates of coverage, contact with experts to identify additional studies, Internet searches) and search date	5
Search	Present full electronic search strategy for at least one database, including any limits used, such that it could be repeated. Search is commonly not driven by a clinical question	Supplementary Appendix p 2
Study selection	Describe the process for selecting studies for inclusion (ie, how many reviewers selected studies, reviewing in duplicate or by single individuals)	5
Data collection process	Describe method of data extraction from reports (eg, piloted forms, independently, in duplicate) and any processes used for manipulating data or obtaining and confirming data from investigators	5,6
Data items	List and define all variables for which data were sought and any assumptions and imputations made	6
Risk of bias in individual studies	If risk of bias assessment of individual studies was relevant to the analysis, describe the items used and how this information is to be used during data synthesis	-
Summary measures	State the principal summary measures (eg, ratio of risk ratios, difference in means) and explain its meaning and direction to readers	6

Synthesis of results	Describe the statistical or descriptive methods of synthesis including measures of consistency if relevant. If applicable, describe the development of statistical or simulation modelling based on theoretical background. Describe and justify assumptions and computational approximations. Describe methods of additional analyses (eg, sensitivity or subgroup analyses, meta-regression), if done, indicating which were prespecified	6
**Results**		
Study selection	Give numbers of studies assessed for eligibility and included in the study, with reasons for exclusions at each stage, ideally with a flow diagram. Present a measure of inter-reviewer agreement (eg, kappa statistic)	-
Study characteristics	For each study, present characteristics for which data were extracted and provide the citations. Clinical characteristics may not always be relevant	-
Risk of bias within studies	If risk of bias assessment of individual studies was used in the meta-epidemiological analysis, report risk of bias indicators of each study to allow replication of findings	-
Results of individual studies	Present data elements used in the meta-epidemiological analysis from each study (results of clinical outcomes may not be relevant)	-
Synthesis of results	Present results of statistical analysis done, including measures of precision and measures of consistency. Present validity of assumptions and fit of statistical or simulation modelling, if applicable	-
Additional analyses	Additional analyses, if done (e.g., sensitivity or subgroup analyses, metaregression)	-
**Discussion**		
Summary of evidence	Summarise the main findings and compare them with existing knowledge about the topic. The quality of evidence may not be relevant; however, investigators should describe their certainty in the results to readers	7
Limitations	Discuss limitations at research methodology level (eg, likelihood of reporting or publication bias	7
Conclusions	Provide general interpretation of the results and implications for future research. Provide any plausible impact on clinical practice	7,8
**Funding**	Describe sources of funding for the methodology research and role of funders	8

5. REFERENCES

1. Pitre T, Cheng S, Cusano E, Khan N, Mikhail D, Leung G, Vernooij RWM, Yarnell CJ, Goligher E, Murthy S, Heath A, Mah J, Rochwerg B, Zeraatkar D. Methodology and design of platform trials: a meta-epidemiological study. J Clin Epidemiol 2023; 157: 1–12. doi: 10.1016/J.JCLINEPI.2023.02.010

2. Higgins JPT, Thomas J, Chandler J et al. (editors). Cochrane Handbook for Systematic Reviews of Interventions version 6.5 (updated August 2024). Chapter 4.4.7. Search Filters. Available from: www.training.cochrane.org/handbook. Cochrane 2024. Accessed March 26, 2025.

3. Angus DC, Alexander BM, Berry S, Buxton M, Lewis R, Paoloni M, Webb SAR, Arnold S, Barker A, Berry DA, Bonten MJM, Brophy M, Butler C, Cloughesy TF, Derde LPG, Esserman LJ, Ferguson R, Fiore L, Gaffey SC, Gaziano JM, Giusti K, Goossens H, Heritier S, Hyman B, Krams M, Larholt K, LaVange LM, Lavori P, Lo AW, London AJ, Manax V, McArthur C, O'Neill G, Parmigiani G, Perlmutter J, Petzold EA, Ritchie C, Rowan KM, Seymour CW, Shapiro NI, Simeone DM, Smith B, Spellberg B, Stern AD, Trippa L, Trusheim M, Viele K, Wen PY, Woodcock J. Adaptive platform trials: definition, design, conduct and reporting considerations. Nat Rev Drug Discov 2019; 18: 797–807. doi: 10.1038/S41573-019-0034-3

4. Shamseer L, Moher D, Clarke M, Ghersi D, Liberati A, Petticrew M, Shekelle P, Stewart LA, Altman DG, Booth A, Chan AW, Chang S, Clifford T, Dickersin K, Egger M, Gøtzsche PC, Grimshaw JM, Groves T, Helfand M, Higgins J, Lasserson T, Lau J, Lohr K, McGowan J, Mulrow C, Norton M, Page M, Sampson M, Schünemann H, Simera I, Summerskill W, Tetzlaff J, Trikalinos TA, Tovey D, Turner L, Whitlock E. Preferred reporting items for systematic review and meta-analysis protocols (PRISMA-P) 2015: elaboration and explanation. BMJ 2015 Jan 2; 350. doi: 10.1136/BMJ.G7647

5. Murad MH, Wang Z. Guidelines for reporting meta-epidemiological methodology research. Evid Based Med 2017; 22: 139–42. doi: 10.1136/EBMED-2017-110713
